# Gene expression in cultured primordial shoots of adult white spruce (*Picea glauca*) in somatic embryogenesis responsive and non responsive genotypes

**DOI:** 10.1186/1753-6561-5-S7-P140

**Published:** 2011-09-13

**Authors:** Krystyna Klimaszewska, Brian Boyle, Sebastien Caron, Don Stewart, Catherine Overton, John MacKay, Robert Rutledge

**Affiliations:** 1 1Natural Resources Canada, Canadian Forest Service, Laurentian Forestry Centre, 1055 du P.E.P.S., Québec, QC G1V 4C7, Canada; 2 2Arborea Project, Centre d’études de la Forêt, Institut de Biologie Intégrative et des Systèmes, Université Laval, 1030 Rue de la Médecine, Québec, QC, G1V 0A6, Canada

## Background

Modern forest management relies on extensive breeding and reforestation programs to sustain forest productivity and conservation of natural forests. Plantation forestry, with its increased productivity and improved wood quality, is likely to become an important source of wood products in the future. Vegetative propagation of superior coniferous forest trees through tissue culture has the potential to deliver a stable supply of superior seedlings for forest plantation. To clone adult (mature) conifer trees by means of tissue culture has been a cherished goal for the last several decades. The benefits derived from improving the genetic make-up of planting stock would be significant if such clonal propagation could be achieved at a high efficiency and without growth abnormalities of regenerated plants. Although, somatic embryogenesis (SE) technology has worked well for many conifer species using zygotic embryos as starting material, attempts to achieve the same in adult conifers have failed. The basis of this failure is not exactly understood.

## Results and discussion

Recently, we have been successful in inducing SE from primordial shoots (PS) of 10 years-old somatic white spruce, genotype 893-6 [1]. We have also identified a few genes (VP1, WOX2, CHAP3A, SAP2C) that were expressed exclusively or significantly up regulated in embryonal mass (EM). In an attempt to gain some understanding of the underlying general molecular events occurring during SE induction phase, we used microarray technology to examine gene expression in responding PS of 893-6 genotype and in non responding PS of 893-12 genotype. Shoot buds were collected from trees in early spring. Some of them were immediately frozen in liquid nitrogen (day 0) and others were disinfected. PS were excised, subdivided into two or four sections and cultured on a standard medium that is used for SE induction from zygotic embryos of white spruce [1]. After 3, 7, 15 and 21 days of culture the PS explants were collected and frozen in liquid nitrogen. RNA was extracted from shoot bud samples at day 0 and from PS explants after 7 days of culture. cDNA was then subjected to microarray analysis using an oligo-based microarray developed by the Arborea project that contains 32,000 probes.

Microarray analysis lead to identification of a number of genes that was up-regulated in each genotype (1-45 fold) in response to day 7 of culture on SE induction medium. Absolute quantification qPCR of the four most up-regulated genes in each genotype confirmed the microarray results, although the magnitude of up-regulation determined by qPCR tended to be greater than that predicted by microarray analysis. In order to provide a more detailed perspective into the dynamics of gene expression, qPCR analysis of these eight genes was expanded to include 3, 15 and 21 days samples. This revealed that while differences in the magnitude of up-regulation for most of these genes differed, the overall trend in expression dynamics were very similar for both genotypes. The one exception was a gene of unknown function, which was rapidly induced in the responsive genotype, reaching a maximum at day 3 followed by progressive reduction over the remaining 18 days. In contrast, expression of this gene remained very low in the non-responsive genotype throughout the entire culture period. The expression level for three other genes peaked between 7-15 days, followed by a reduction at 21 days in both genotypes, whereas the remaining four genes showed a progressive increase up to the last day in culture in both genotypes.This project also included analysis of nine candidate reference genes, which demonstrated that expression of five were highly stable (<±20% CV).
